# Assembly and comparative analysis of the complete mitochondrial genome of *Suaeda glauca*

**DOI:** 10.1186/s12864-021-07490-9

**Published:** 2021-03-09

**Authors:** Yan Cheng, Xiaoxue He, S. V. G. N. Priyadarshani, Yu Wang, Li Ye, Chao Shi, Kangzhuo Ye, Qiao Zhou, Ziqiang Luo, Fang Deng, Ling Cao, Ping Zheng, Mohammad Aslam, Yuan Qin

**Affiliations:** 1 State Key Laboratory of Ecological Pest Control for Fujian and Taiwan Crops, College of Plant Protection, Fujian Provincial Key Laboratory of Haixia Applied Plant Systems Biology, Center for Genomics and Biotechnology, College of Life Science, Fujian Agriculture and Forestry University, Fuzhou, 350002 China; 2 College of Agriculture, Fujian Agriculture and Forestry University, Fuzhou, China; 3 State Key Laboratory for Conservation and Utilization of Subtropical Agro-Bioresources, Guangxi Key Lab of Sugarcane Biology, College of Agriculture, Guangxi University, Nanning, 530004 Guangxi China

**Keywords:** *Suaeda glauca*, Mitochondrial genome, Repeats, Phylogenetic analysis

## Abstract

**Background:**

*Suaeda glauca* (*S. glauca*) is a halophyte widely distributed in saline and sandy beaches, with strong saline-alkali tolerance. It is also admired as a landscape plant with high development prospects and scientific research value. The *S. glauca* chloroplast (cp) genome has recently been reported; however, the mitochondria (mt) genome is still unexplored.

**Results:**

The mt genome of *S. glauca* were assembled based on the reads from Pacbio and Illumina sequencing platforms. The circular mt genome of *S. glauca* has a length of 474,330 bp. The base composition of the *S. glauca* mt genome showed A (28.00%), T (27.93%), C (21.62%), and G (22.45%). *S. glauca* mt genome contains 61 genes, including 27 protein-coding genes, 29 tRNA genes, and 5 rRNA genes. The sequence repeats, RNA editing, and gene migration from cp to mt were observed in *S. glauca* mt genome. Phylogenetic analysis based on the mt genomes of *S. glauca* and other 28 taxa reflects an exact evolutionary and taxonomic status of *S. glauca*. Furthermore, the investigation on mt genome characteristics, including genome size, GC contents, genome organization, and gene repeats of *S. gulaca* genome, was investigated compared to other land plants, indicating the variation of the mt genome in plants. However, the subsequently Ka/Ks analysis revealed that most of the protein-coding genes in mt genome had undergone negative selections, reflecting the importance of those genes in the mt genomes.

**Conclusions:**

In this study, we reported the mt genome assembly and annotation of a halophytic model plant *S. glauca.* The subsequent analysis provided us a comprehensive understanding of the *S. glauca* mt genome, which might facilitate the research on the salt-tolerant plant species.

**Supplementary Information:**

The online version contains supplementary material available at 10.1186/s12864-021-07490-9.

## Background

*Chenopodiaceae* is among the large families of angiosperms that mainly include *Spinacia oleracea*, *Chenopodium quinoa Willd*, and *Beta vulgaris* [1–3]. *Chenopodiaceae* plants are mostly annual herbs, half shrubs, shrubs, living in the desert, and saline soil areas. Therefore, they often show xerophytic adaptation. As an annual herb of *Chenopodiaceae*, *S. glauca* grows in saline-alkali land and beaches. It displays a strong salt tolerance and drought tolerance capacity and has high value as medicine and food material [4–6]. Moreover, *S. glauca* possesses immense ecological importance as it can tolerate heavy metals at higher levels and could be used as a super accumulator of heavy metals. The environmental protection and remediation of contaminated soil make it a natural resource with significant economic and ecological importance [7].

Plant mt is involved in numerous metabolic processes related to energy generation and the synthesis and degradation of several compounds [8]. Margulis’ endosymbiosis theory suggests that mt originated from archaea living in nucleated cells when eukaryotes swallowed the bacteria. Later it evolved into organelles with special functions during the long-term symbiosis [9–11], incorporated as an additional mt genome. Mitochondria convert biomass energy into chemical energy through phosphorylation and provide energy for life activities. Besides, it is involved in cell differentiation, apoptosis, cell growth, and cell division [12–15]. Therefore, mitochondria play a crucial role in plant productivity and development [16]. For most seed plants, nuclear genetic information is inherited from both parents, while cp and mt are inherited from the maternal parent. This genetic mechanism eliminates the paternal lines’ influence, thus reducing the difficulty of genetic research and facilitating the study of genetic mechanisms [17].

With the development of sequencing technology, an increasing number of mt genomes have been reported. Up to Jan. 2021, 351 complete mt genomes have been deposited in GenBank Organelle Genome Resources. Long periods of mutualism leave mitochondria with some of their original DNA lost, and some of them transferred, leaving only the DNA that codes for it [18, 19]. Mt DNA has long been recognized as tending to integrate DNA from various sources through intracellular and horizontal transfer [20]. Therefore, the mt genome in plants has significant differences in length, gene sequence, and gene content [21]. The mt genome length of the smallest known terrestrial plant is about 66 Kb, and the largest terrestrial plant mt genome length is 11.3 Mb [22, 23]. As a result, the amount of genes in terrestrial plants varies widely, typically between 32 and 67 [24]. In this study, we sequenced and annotated the mt genome of *S. glauca* and compared it with the genomes of other angiosperms (as well as gymnosperms), which provides additional information for a better understanding of the genetics of the halophyte *S. glauca*.

## Results

### Genomic features of the *S. glauca* mt genome

**Fig. 1:**
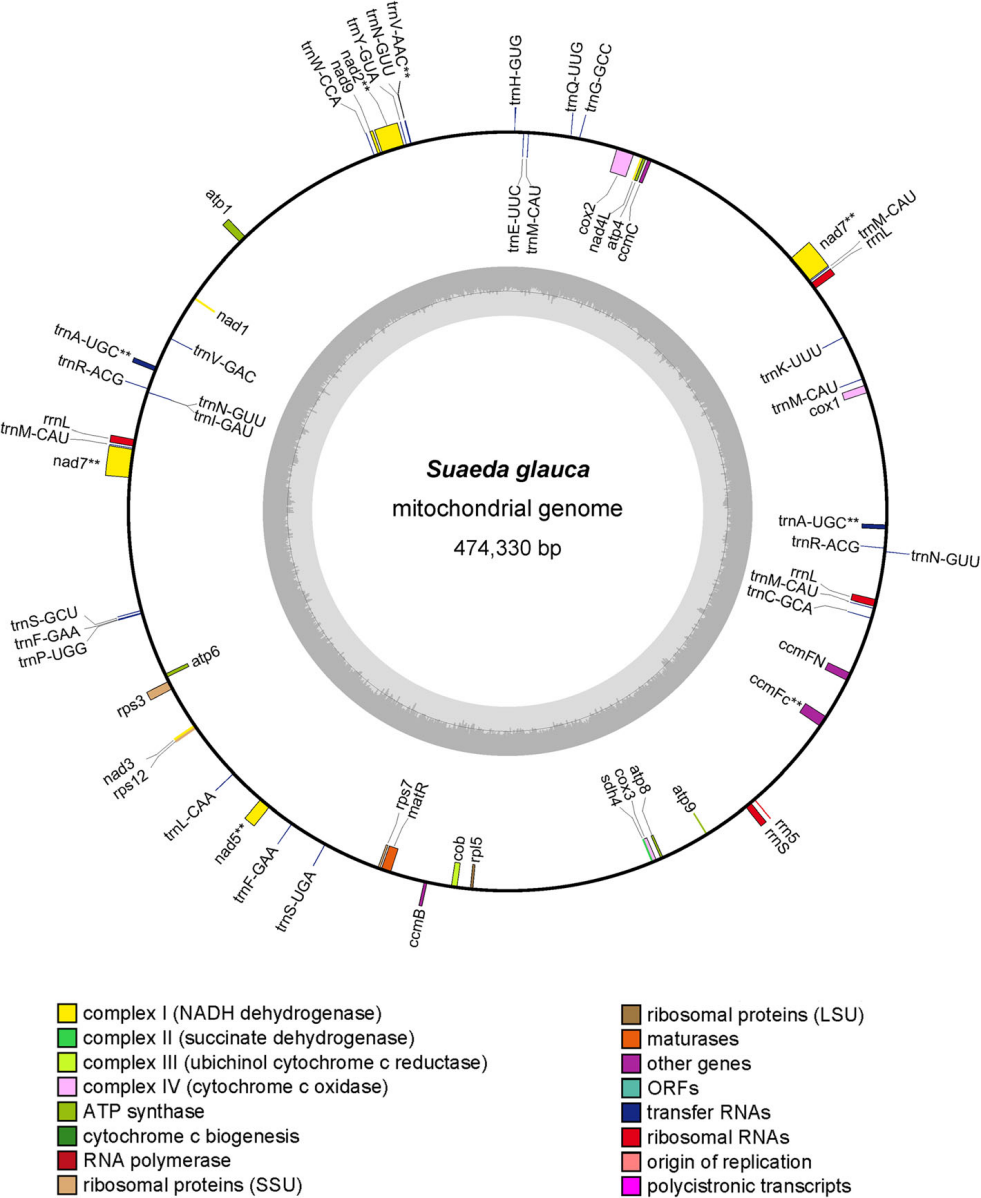
The circular map of *S. glauca* mt genome*.* Gene map showing 61 annotated genes of different functional groups

The *S. glauca* mt genome is circular with a length of 474,330 bp. The base composition of the genome is A (28.00%), T (27.93%), C (21.62%), G (22.45%). There are 61 genes annotated in the mt genome, including 27 protein-coding genes, 29 tRNA genes, and 5 rRNA genes. The functional categorization and physical locations of the annotated genes were presented in Fig. 1. According to our findings, the mt genome of *S. glauca* encodes 26 different protein (*nad7* has two copies) that could be divided into 9 classes (Table 1): NADH dehydrogenase (7 genes), ATP Synthase (5 genes), Cytochrome C Biogenesis (4 genes), Cytochrome C oxidase (3 genes), Ribosomal proteins (SSU) (3 genes), Ribosomal proteins (LSU) (1 gene), Transport membrane protein (1 gene), Maturases (1 gene), and Ubiquinol Cytochrome c Reductase (1 gene). The homologs of *S. glauca* mt genes in the mt genomes of *H. sapiens*, *S. cerevisiae,* and *A. thaliana* were identified and listed in Table S1. All of the protein-coding genes used ATG as starting codon, and all three stop codons TAA, TGA, and TAG were found with the following utilization rate: TAA 44.4%, TGA 37.04%, and TAG 18.52% (Table S2). It is reported that the mt genomes of land plants contain variable number of introns [25]. In the mt genome of *S. glauca*, there are 8 intron-containing genes (*nad2, nad5, nad7* with two copyies, *cox2, ccmFc, trnA-UGC, and trnV-AAC*) harboring 15 introns in total with a total length of 16,743 bp. The intron lengths varied from 105 bp (*trnV-AAC*) to 2103 bp (*nad2*). The gene *nad7* has two copies in the mt genome, and each copy contains 4 introns, which is the highest intron number. The *trnV-AAC*, instead, contains only one intron with a length of 105 bp, which is the smallest intron.

**Table 1:** Gene profile and organization of *S. glauca* mt genome

Group of genes	Gene name	Length	Start codon	Stop codon	Amino acid
**NADH dehydrogenase**	*nad1*	327	ATG	TGA	108
	*nad2*^*a*^	915	ATG	TAA	304
	*nad3*	357	ATG	TAA	118
	*nad4L*	273	ATG	TAA	90
	*nad5*^*a*^	1452	ATG	TGA	483
	*nad7* ^*a*^ (2)	1092	ATG	TAG	363
	*nad9*	579	ATG	TAA	192
**ATP synthase**	*atp1*	1521	ATG	TAA	506
	*atp4*	597	ATG	TAG	198
	*atp6*	741	ATG	TAA	246
	*atp8*	480	ATG	TGA	159
	*atp9*	240	ATG	TGA	79
**Cytochrome c biogenesis**	*ccmB*	621	ATG	TGA	206
	*ccmC*	744	ATG	TAA	247
	*ccmFC*^*a*^	1338	ATG	TAG	445
	*ccmFN*	1635	ATG	TGA	544
**Cytochrome c oxidase**	*cox1*	1575	ATG	TAA	524
	*cox2*^*a*^	768	ATG	TAA	255
	*cox3*	798	ATG	TGA	265
**Maturases**	*matR*	1968	ATG	TAG	655
**Ubiquinol cytochrome c reductase**	*cob*	1182	ATG	TGA	393
**Ribosomal proteins (LSU)**	*rpl5*	555	ATG	TAA	184
**Ribosomal proteins (SSU)**	*rps3*	1680	ATG	TAA	559
	*rps7*	447	ATG	TAA	148
	*rps12*	381	ATG	TGA	126
**Transport membrane protein**	*sdh4*	294	ATG	TGA	97
**Ribosomal RNAs**	*rrn5*	119			
	*rrnS*	1303			
	*rrnL (3)*	1369			
**Transfer RNAs**	*trnA-UGC*^*a,b*^ *(2)*	(73, 73)			
	*trnC-GCA*	76			
	*trnE-UUC*	72			
	*trnF-GAA (2)*	(74, 74^*b*^)			
	*trnG-GCC*	74			
	*trnH-GUG*^*b*^	76			
	*trnI-GAU*^*b*^	79			
	*trnK-UUU (2)*	(73,73)			
	*trnL-CAA*	83			
	*trnM-CAU (4)*	(74^*b*^,76,76,76)			
	*trnN-GUU (3)*	(74^*b*^,74^*b*^,74)			
	*trnP-UGG*	90			
	*trnQ-UUG*	72			
	*trnR-ACG* ^*b*^ *(2)*	(75,75)			
	*trnS-GCU*	91			
	*trnS-UGA*	88			
	*trnV-GAC*^*b*^	72			
	*trnV-AAC*^*a*^	94			
	*trnW-CCA*	74			
	*trnY-GUA*	84			

Notes: The numbers after the gene names indicate the duplication number. Lowercase a indicates the genes containing introns, and lowercase b indicates the cp-derived genes

It has been reported that most land plants contain 3 rRNA genes [9, 11]. Consistently, three rRNA genes *rrn5* (119 bp), *rrnS* (1303 bp), and *rrnL* (1369 bp) were annotated in *S. glauca* mt genome. Besides, 20 different transfer RNAs were identified in *S. glauca* mt genome transporting 18 amino acids, since more than one transfer RNAs might transport the same amino acid for different codons. For example, *trnS-UGA* and *trnS-GCU* transport Ser for synonymous codons UCA and AGC, respectively. Moreover, we observed that transfer RNA *trnF-GAA, trnM-CAU*, and *trnN-GUU* have two different structures with the same anticodon. Taking *trnM-CAU* as an example, both A and B structures share the same anticodon CAU transporting amino acid Met (Figure S1).

### Repeat sequences anaysis

**Fig. 2:**
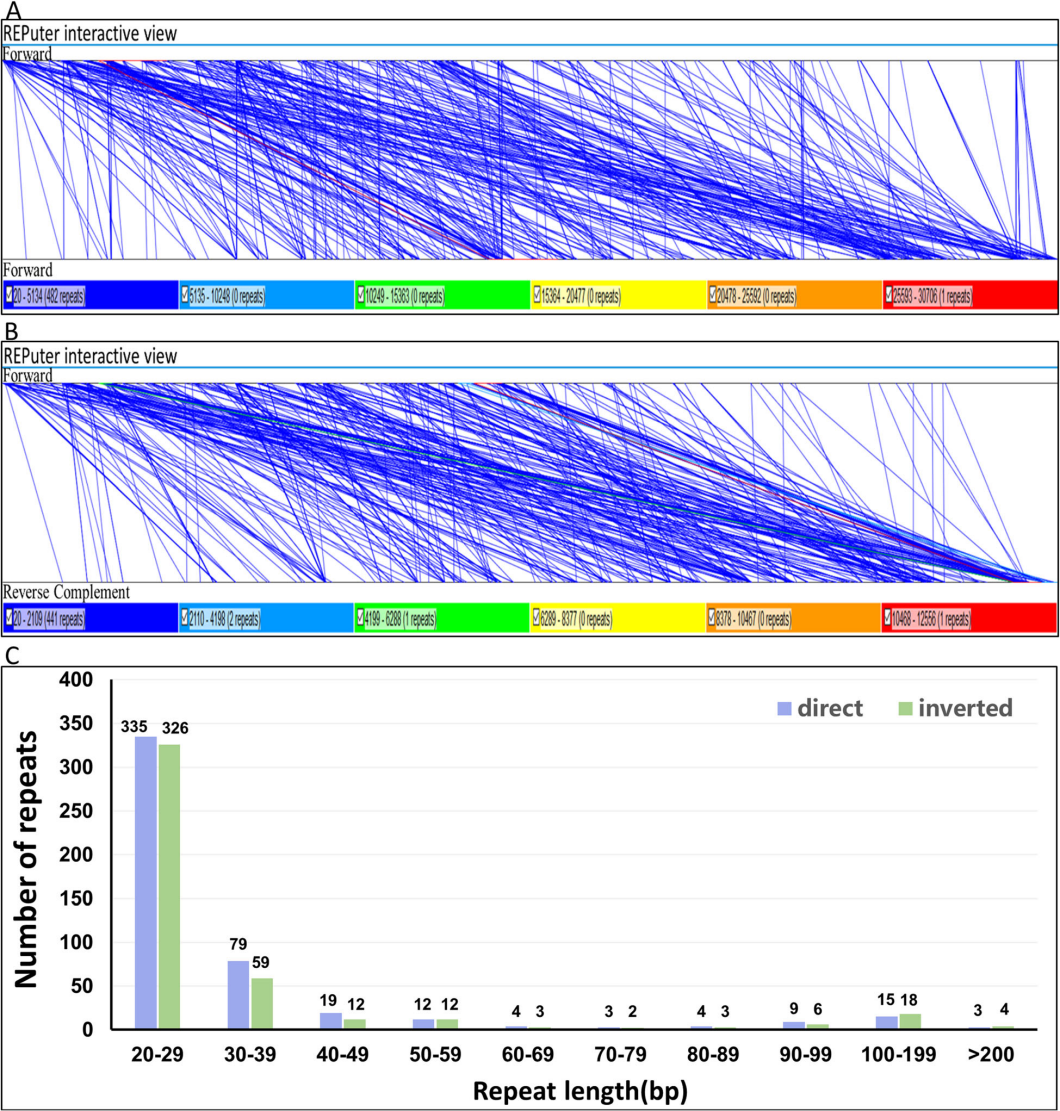
The repeats in *S. glauca* mt genome. **a** The synteny between the mt genome and its forward copy showing the direct repeats. **b** The synteny between the mt genome and its reverse complementary copy showing the inverted repeats. **c** The length distribution of reverse and inverted repeats in *S. glauca* mt genome. The number on the histograms represents the repeat number of designated lengths shown on the horizontal axis

Microsatellites, or simple sequence repetitions (SSRs), are DNA fragments consisting of short units of sequence repetition of 1–6 base pairs in length [26]. The uniqueness and the value of microsatellites are due to their polymorphism, codominant inheritance, relative abundance, extensive genome coverage, and simplicity in PCR detection [27]. SSRs in the mt genome of *S. glauca* were identified with Tandem Repeats Finder software [28]. As a result, 361 SSRs were found in the mt genome of *S. glauca*, and the proportion of different forms were shown in Figure S2. SSRs in monomer and dimer forms accounted for 78.67% of the total SSRs present. Adenine (A) monomer repeats represented 46.28% (56) of 121 monomer SSRs, and AT repeat was the most frequent type among the dimeric SSRs, accounting for 58.15%. There are only two hexameric SSRs presented in *S. glauca* mt genome, located between *nad4L* and *cox2,* and between *trnQ-UUG* and *trnM-CAU*. The specific locations of pentamer and hexamer are shown in Table 2. Tandem repeats, also named satellite DNA, refer to the core repeating units of about 1 to 200 bases, repeated several times in tandem. They are widely found in eukaryotic genomes and in some prokaryotes [29]. As shown in Table 3, a total of 12 tandem repeats with a matching degree greater than 95% and a length ranging from 13 bp to 38 bp were present in the mt genome of *S. glauca*. The non-tandem repeats in *S. glauca* mt genome were also detected using REPuter software [30]. As a result, 928 repeats with the length equal to or longer than 20 were observed, of which 483 were direct, and 445 were inverted. The longest direct repeat was 30,706 bp, while the longest inverted repeat was 12,556 bp (Supplementary data sheet 1). The length distribution of the direct and inverted repeats are shown in Fig. 2. It is shown that the 20–29 bp repeats are most abundant for both repeat types.

**Table 2:** Distribution of penta and hexa SSRs in *S. glauca* mt genome

No.	Type	SSR	Start	End	Location
1	pentamer	(tatac) × 3	3006	3020	*cox1*
2	pentamer	(agaat) × 3	49,581	49,595	*nad7*
3	pentamer	(taagt) × 3	78,725	78,739	IGS (*nad7,trnI*)
4	pentamer	(ggaaa) × 3	107,921	107,935	IGS (*trnQ-UUG,trnM-CAU*)
5	pentamer	(cgggc) × 3	139,703	139,717	IGS (*nad2,nad9*)
6	pentamer	(cttct) × 3	168,170	168,184	IGS (*trnW-CCA,atp1*)
7	pentamer	(tcttg) × 3	201,546	201,560	IGS (*trnV-GAC,trnA-UGC*)
8	pentamer	(agaat) × 3	225,057	225,071	*nad7*
9	pentamer	(ttctt) × 3	316,091	316,105	IGS *(trnF-GAA.trnS-UGU)*
10	pentamer	(actag) × 3	330,081	330,095	*matR*
11	pentamer	(caaaa) × 3	388,600	388,614	IGS (*atp8,atp9)*
12	pentamer	(agaaa) × 3	401,486	401,500	IGS (*atp9, rrnS)*
13	hexamer	(caaaat) × 3	92,262	92,279	IGS (*nad4L, cox2*)
14	hexamer	(tagaaa) × 3	106,488	106,505	IGS (*trnQ-UUG, trnM-CAU*)

**Table 3:** Distribution of perfect tandem repeats in *S. glauca* mt genome

No.	Size	Repeat sequence	Copy	Percent Matches	Start	End
1	9	TACTGTAGC	4	96	37,660	37,694
	9	TTGTAGTTT	3	100	37,689	37,714
3	32	CCATACTTGTTCCAAGTAAGTGAATTGCATTA	6	99	48,018	48,212
4	31	GAGACAAGTCTAGTATAGACGCAGGGTCGAA	5	98	104,348	104,524
5	38	TTTCGGAAGTTTTATCCTATAAGAATTGGCTTTTCCTT	2	95	168,613	168,711
6	13	TCTAATAGAAAAT	2	100	201,473	201,497
7	16	AATGTGTATTATCCAT	2	100	294,569	294,601
8	18	ATATCGTCACTAGCATCA	2	100	296,770	296,808
9	9	ATCGATGAT	3	100	297,459	297,484
10	18	AGTCTATCAACGCTACTG	2	100	335,715	335,749
11	9	TGAAGTTAT	3	100	394,462	394,486
12	32	GGTAATGCCAATTCACTTACTTGGAACAAGTAT	6	99	454,228	454,422

### The prediction of RNA editing

**Fig. 3:**
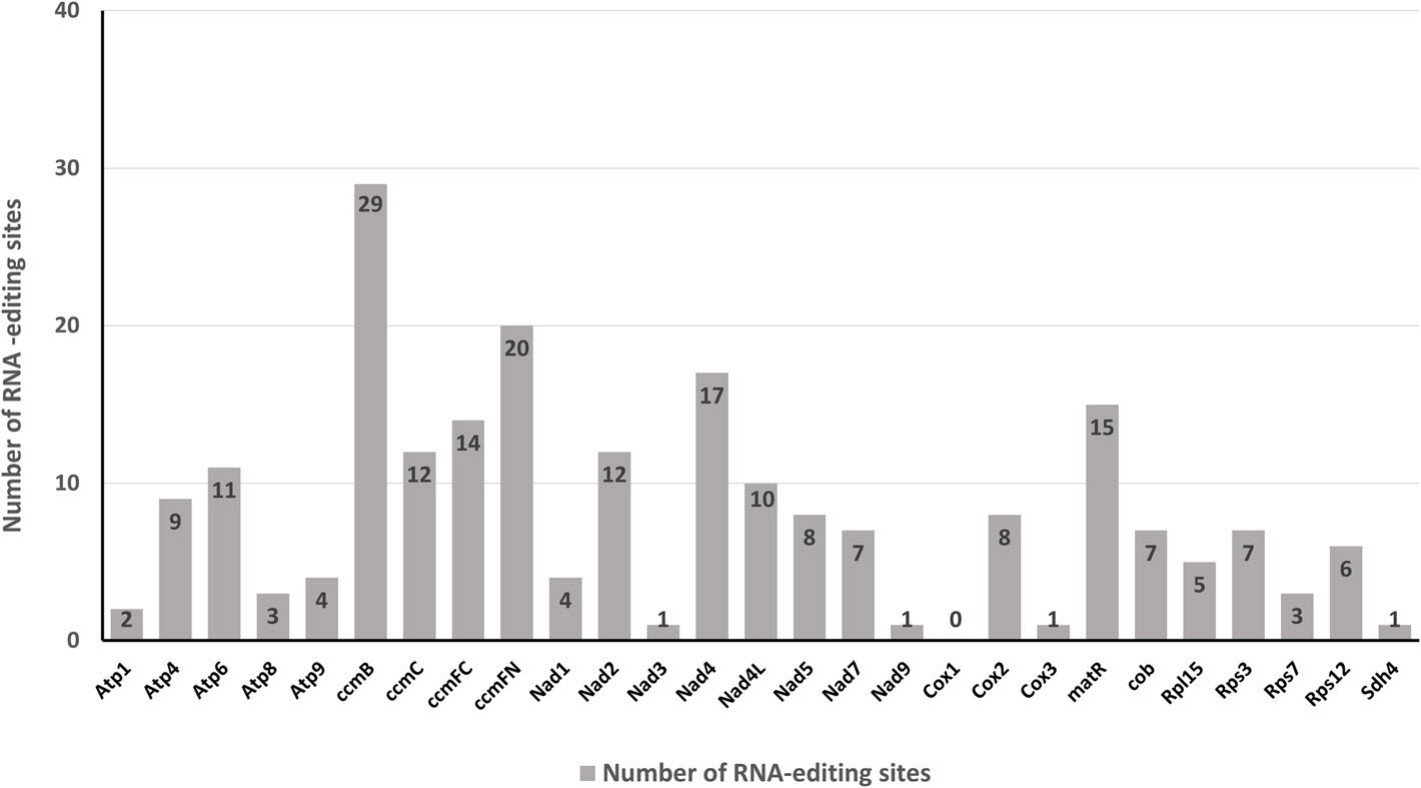
The distribution of RNA-editing sites in *S. glauca* mt protein-coding genes. The gray bars represent the number of RNA-editing sites of each gene

RNA editing refers to the addition, loss, or conversion of the base in the coding region of the transcribed RNA [31], found in all eukaryotes, including plants [32]. In chloroplast and mitochondrion, the conversion of specific cytosine into uridine alters the genomic information [33]. This process improves protein preservation in plants by modifying codons. Without the support of the proteomics data, it is impossible to detect accurate RNA editing. However, Mower’s software PREP could be used to computationally predict the RNA edit site [34]. In this analysis, 216 RNA editing sites within 26 protein-coding genes (Table 4) were predicted in the mt genome of *S. glauca*, using PREP-MT program (Fig. 3). Among those protein-coding genes, *cox1* does not have any editing site predicted, while *ccmB* has the most editing sites predicted (29). Of those editing sites, 35.19% (76) were located at the first position of the triplet codes, 63.89% (138) occurred with the second base of the triplet codes. And there was a particular editing case in which the first and second positions of the triplet codes were edited, resulting in an amino acid change from the original proline (CCC) to phenylalanine (TTC). After the RNA editing, the hydrophobicity of 42.13% of amino acids did not change. However, 45.83% of the amino acids were were predicted to change from hydrophilic to hydrophobic, while 11.11% were predicted to change from hydrophobic to hydrophilic. The RNA editing might lead to the premature termination of protein-coding genes, and this phenomenon is likely to occur with *atp4* and *atp9* in *S. glauca* mt genome. Our results also showed that the amino acids of predicted editing codons showed a leucine tendency after RNA editing, which is supported by the fact that the amino acids of 47.69% (103 sites) of the edits were converted to leucine (Table 4).

**Table 4:** Prediction of RNA editing sites

Type	RNA -editing	Number	Percentage
hydrophobic	CCA (P) = > CTA (L)	20	31.02%
	CCG (P) = > CTG (L)	14	
	CCC (P) = > CTC (L)	7	
	CCT (P) = > CTT (L)	6	
	CCC (P) = > TTC (F)	2	
	GCC (A) = > GTC (V)	3	
	GCG (A) = > GTG (V)	2	
	GCT (A) = > GTT (V)	1	
	GCA (A) = > GTA (V)	1	
	CTT (L) = > TTT (F)	8	
	CTC (L) = > TTC (F)	3	
hydrophilic	CAT (H) = > TAT (Y)	8	11.11%
	CAC (H) = > TAC (Y)	4	
	CGT (R) = > TGT (C)	10	
	CGC (R) = > TGC (C)	2	
hydrophobic-hydrophilic	CCT (P) = > TCT (S)	9	11.11%
	CCA (P) = > TCA (S)	8	
	CCC (P) = > TCC (S)	7	
hydrophilic-hydrophobic	CGG (R) = > TGG (W)	15	45.83%
	TCC (S) = > TTC (F)	11	
	TCT (S) = > TTT (F)	9	
	TCA (S) = > TTA (L)	37	
	TCG (S) = > TTG (L)	19	
	ACC (T) = > ATC (I)	3	
	ACT (T) = > ATT (I)	2	
	ACA (T) = > ATA (I)	1	
	ACG (T) = > ATG (M)	2	
hydrophilic-stop	CAA (Q) = > TAA (X)	1	0.93%
	CGA (R) = > TGA (X)	1

### DNA migration from chloroplast to mitochondria

Thirty-two fragments with a total length of 26.87 kb were observed to be migrated from cp genome to mt genome in *S. glauca*, accounting for 5.18% of the mt genome. There are 8 annotated genes located on those fragments, all of which are tRNA genes, namely *trnA-UGC, trnF-GAA, trnH-GUG, trnI-GAU, trnR-ACG, trnM-CAU, trnN-GUU, and trnV-GAC*. Our data also demonstrate that some chloroplast protein-coding genes, i.e. *atpA, rrn16, rrn23, rpoC2, ndhA, psaB, and psbB* migrated from cp to mitochondrion, even though most of them lost their integrities during evolution, and only partial sequences of those genes could be found in the mt genome nowadays (Table 5). The different destinations of transferred protein-coding genes and tRNA genes suggested that tRNA genes are much more conserved in the mt genome than the protein-coding genes, indicating their indispensable roles in mitochondria.

**Table 5:** Fragments transferred from chloroplast to mitochondria in *S. glauca*

	Alignment length	Identity%	Mismatches	Gap opens	mt start	mt end	cp start	cp end	Gene
1	3954	95.726	82	20	295,697	299,575	90,318	86,377	
2	3527	98.469	16	10	207,557	211,072	99,899	103,398	*trnA-UCG*
3	3527	98.441	16	11	468,275	471,789	128,572	132,071	
4	3142	97.581	18	15	292,489	295,603	93,422	90,312	*trnI-GAU*
5	2545	96.149	35	19	465,776	468,283	126,021	128,539	*trnN-GUU,* *trnR-ACG*
6	2546	95.915	39	24	211,064	213,571	103,431	105,949	
7	2031	99.015	8	2	199,446	201,472	133,093	135,115	*trnV-GAC*
8	1063	93.509	20	12	201,516	202,548	96,852	95,809	
9	533	94.934	21	4	310,145	310,671	47,809	48,341	*trnF-GAA*
10	427	97.424	11	0	246,135	246,561	33,914	33,488	
11	427	97.424	11	0	70,659	71,085	33,914	33,488	
12	388	96.392	14	0	370,829	371,216	19,553	19,940	
13	351	95.442	16	0	438,325	438,675	118,358	118,008	*ndhA*^*a*^
14	279	95.341	13	0	307,665	307,943	71,873	71,595	*psbB*^*a*^
15	248	93.952	15	0	14,593	14,840	10,031	9784	*atpA*^*a*^
16	888	73.649	181	39	407,200	408,058	97,374	98,237	*rrn16*^*a*^
17	157	98.726	2	0	404,203	404,359	42,089	41,933	
18	289	85.121	16	10	309,891	310,153	46,981	47,268	
19	340	79.706	48	15	145,392	145,717	64,797	64,465	*trnW-CCA*^*b*^
20	111	96.396	4	0	349,247	349,357	97,265	97,155	
21	86	96.512	3	0	138,006	138,091	105,510	105,425	*trnN-GUU*
22	78	97.436	2	0	117,112	117,189	79	2	*trnH-GUG*
23	77	96.104	2	1	309,789	309,865	46,566	46,641	
24	76	93.421	5	0	114,384	114,459	51,243	51,168	*trnM-CAU*
25	79	92.405	6	0	353,124	353,202	141,760	141,838	
26	56	98.214	1	0	248,777	248,832	37,491	37,436	*psaB*^*a*^
27	56	98.214	1	0	73,301	73,356	37,491	37,436	
28	45	97.778	1	0	274,465	274,509	16,239	16,195	*rpoC2*^*a*^
29	42	97.619	1	0	239,555	239,596	101,136	101,095	*rrn23*^*a*^
30	42	97.619	1	0	64,079	64,120	130,834	130,875	
31	42	97.619	1	0	239,555	239,596	130,834	130,875	
32	61	88.525	4	3	353,019	353,077	96,110	96,169	
Total	27,513								

Notes: Lowercase a indicates the partial sequence found in mt genome. Lowercase b indicates the mt-derived genes

### Phylogenetic analysis within higher plant mt genomes

**Fig. 4:**
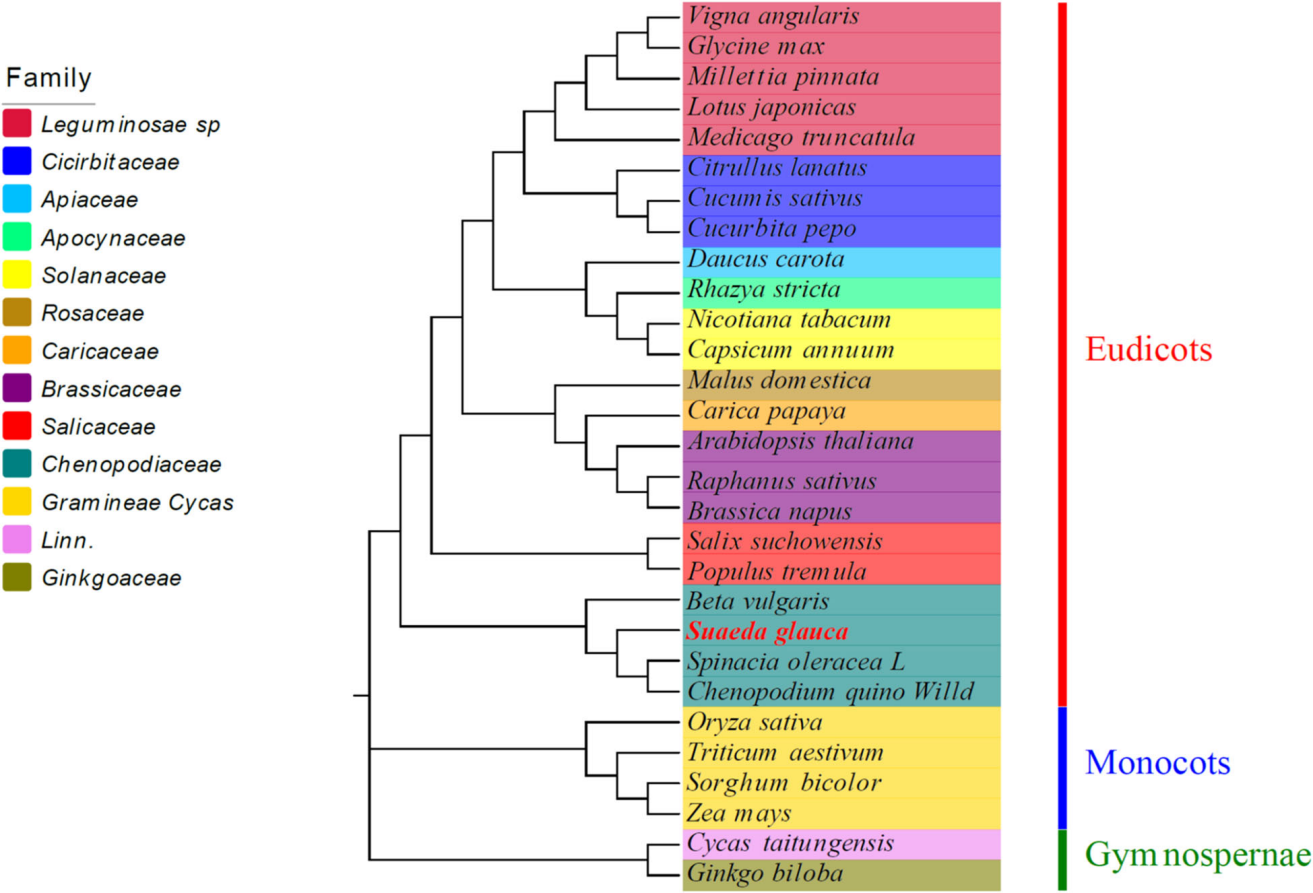
The phylogenetic relationships of *S. glauca* with other 28 plant species. The Neighbor-Joining tree was constructed based on the sequences of 23 conserved protein-coding genes. Colors indicate the families that the specific species belongs

To understand the evolutionary status of *S. glauca* mt genome, the phylogenetic analyses was performed on *S. glauca* together with other 28 species, including 22 eudicots, 4 monocots, and 2 gymnosperms (designated as outgroups). Abbreviations and the accession number of mt genomes investigated in this study are listed in Table S3. A phylogenetic tree was obtained based on an aligned data matrix of 23 conserved protein-coding genes from these species, as shown in Fig. 4. The phylogenetic tree strongly supports the separation of eudicots from monocots and the separation of angiosperms from gymnosperms. Moreover, the taxa from 13 families (*Leguminosae*, *Cucurbitaceae*, *Apiaceae*, *Apocynaceae*, *Solanaceae*, *Rosaceae*, *Caricaceae*, *Brassicaceae*, *Salicaceae*, *Chenopodiaceae*, *Gramineae*, *Cycadaceae*, and *Ginkgoaceae*) were well clustered. The order of taxa in the phylogenetic tree was consistent with the evolutionary relationships of those species, indicating the consistency of traditional taxonomy with the molecular classification. Based on the phylogenetic relationships among the 29 species, different groups of plants were selected for further comparative analysis.

### The comparison of mt genome size and GC content between *S. glauca* and other species

**Fig. 5:**
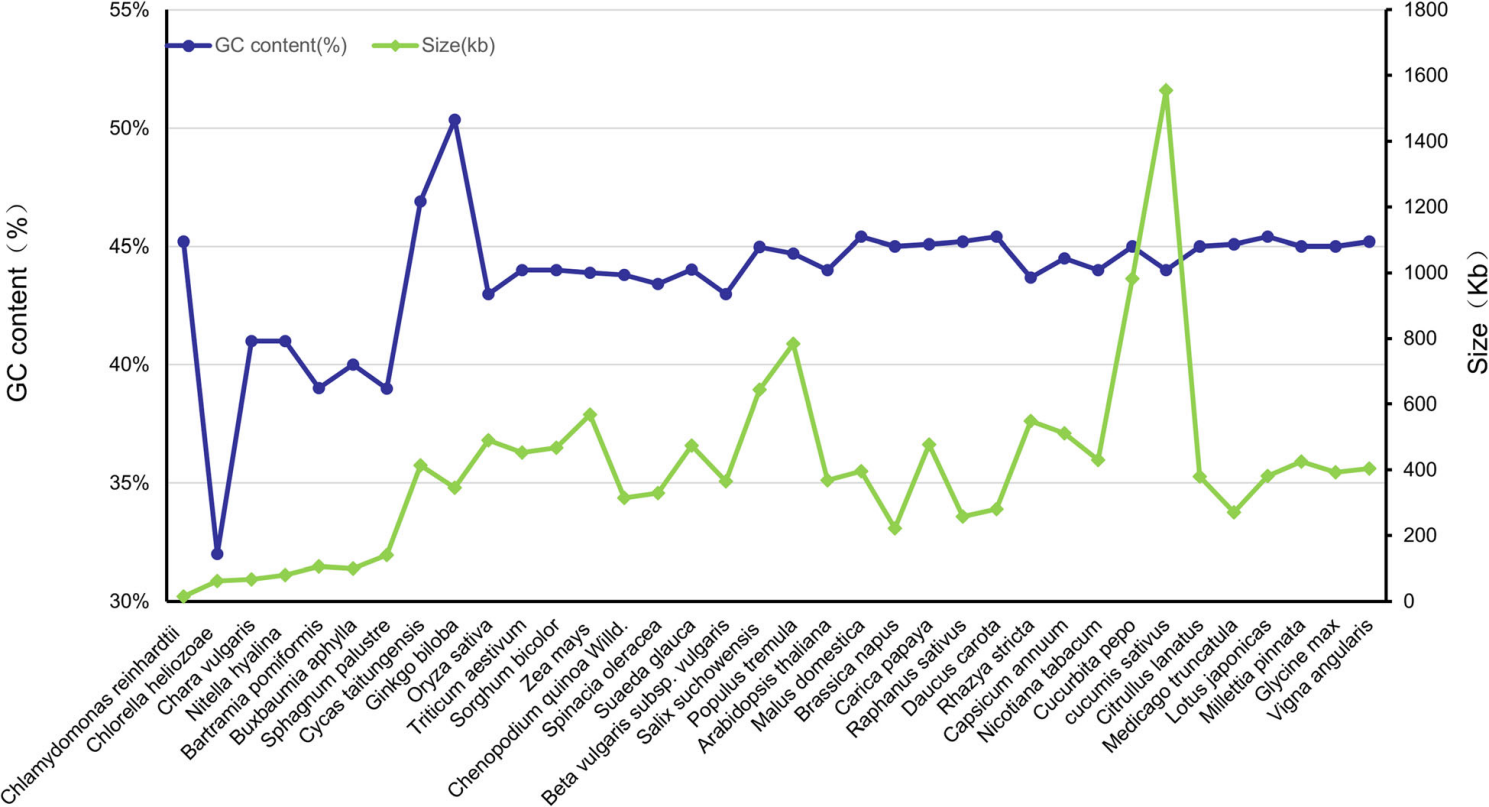
The sizes and GC contents of 36 mt plant genomes. The blue dots represent the GC content of the taxa, and the blue trendline shows the variation of GC content across the different taxa. The green dots represent the genome size, and the trendline shows the variation of GC content

The size and GC content are the primary characteristics of an organelle genome. We compared the size and GC content of *S. glauca* with other 35 green plants, including 4 phycophyta, 3 bryophytes, 2 gymnosperms, 4 monocots, and 22 dicots. The abbreviations of species names of those plants and the accession numbers of their mt genomes are listed in Table S3. As shown in Fig. 5, the sizes of mt genomes varied from 15,758 bp (*C. reinhardtii*) to 1,555,935 bp (*C. sativus*). The sizes of mt genomes of phycophyta and bryophytes were generally smaller compared to land plants, while that of *S. glauca* (474,330 bp) has an average size. Similarly, the GC contents of the mt genomes were also variable, ranging from 32.24% in *S. palustre* to 50.36% in *G. biloba*. In general, the GC contents of angiosperms, including monocots and dicots, are larger than those of bryophytes but smaller than those of gymnosperms, suggesting that the GC contents frequently changed after the divergence of angiosperms from bryophytes and gymnosperms. Interestingly, our results also showed that the GC contents fluctuate widely in phycophyta. In contrast, the GC contents in angiosperms were much conserved during the evolution, although their genome sizes varied tremendously.

### Comparison of genome organization with ten green plant mt genomes

The *S. glauca* mt genome organization was extensively investigated for protein-coding genes, cis-spliced introns, rRNAs tRNAs, and non-coding regions. It was further compared with 10 other taxa, including 3 plants from *Chenopodiaceae*. As shown in Table 6, protein-coding genes and cis-introns regions represent 5.00% and 3.92% of the whole *S. glauca* mt genome sequence, respectively. In comparison, the proportions of rRNA and tRNA regions represent only 1.17% and 0.47%, respectively. The other three plants from *Chenopodiaceae* have similar proportions of protein-coding genes, slightly higher than that of *S. glauca*. However, the proportions of coding regions were significantly different across families, probably due to the different mt genome sizes.

**Table 6:** Organization of mt genomes of *S. glauca* and other ten green plants

Plant species	Family	Coding regions (%)				Non-coding regions (%)
		Protein-coding genes	Cis-spliced introns	rRNAs	tRNAs	
*G. biloba*	*Ginkgoaceae*	9.95	11.31	1.44	0.50	76.80
*Z. mays*	*Gramineae*	6.06	4.06	0.99	0.28	88.61
*B. vulgaris*	*Chenopodiaceae*	7.63	3.62	3.30	0.54	84.90
*C. quinoa Willd.*	*Chenopodiaceae*	8.47	4.89	1.71	0.51	84.43
*S. oleracea*	*Chenopodiaceae*	8.37	5.69	1.64	0.52	83.79
*S. glauca*	*Chenopodiaceae*	5.00	3.92	1.17	0.47	89.44
*S. suchowensis*	*Salicaceae*	4.68	4.21	0.83	0.27	90.01
*A. thaliana*	*Brassicaceae*	8.53	7.99	1.42	0.54	81.52
*N. tabacum*	*Solanaceae*	7.11	14.47	2.05	0.40	76.00
*C. papaya*	*Caricaceae*	7.12	6.27	1.14	0.30	85.17
*G. max*	*Leguminosae*	8.48	8.09	1.31	0.35	81.77

### Gene duplication and lost in mt genomes of *Chenopodiaceae *plants

With the rapid development of sequencing technology, an increasing number of complete plant mt genomes were assembled and reported recently, facilitating the comparison analysis of the mt genome features among multiple plant species [35]. As described by Richardson et al., the mt genomes in plants vary considerably in size, gene content, and gene order [21]. The *Chenopodiaceae* plants have a relatively strong tolerance to biotic stress, especially to salt. Four mt genomes from this family: *C. quinoa willd*, *S. oleracea*, *B. vulgaris*, and *S. glauca* are already available. To understand whether those four plants have the same gene contents, the protein-coding genes from those 4 mt genomes were compared. As shown in Table S4, the specific gene duplication and gene loss were observed in different species. For example, *nad7* was duplicated in *S. glauca* mt genome, and *nad1* and *rps7* were duplicated in *B. vulgaris* mt genome. The *C. quinoa* has the most intact mt genome, with only one gene (*sdh4*) loss, while *atp4* and *ccmC* from *B. vulgaris ssp*, and *nad1* and *shh4* from *S. oleracea* were also lost. However, with five genes, *nad4*, *nad6*, *rps4*, *rps13*, and *tatC,* gene loss appears more frequent in the mt genome of *S. glauca*.

### The substitution rates of protein-coding genes

**Fig. 6:**
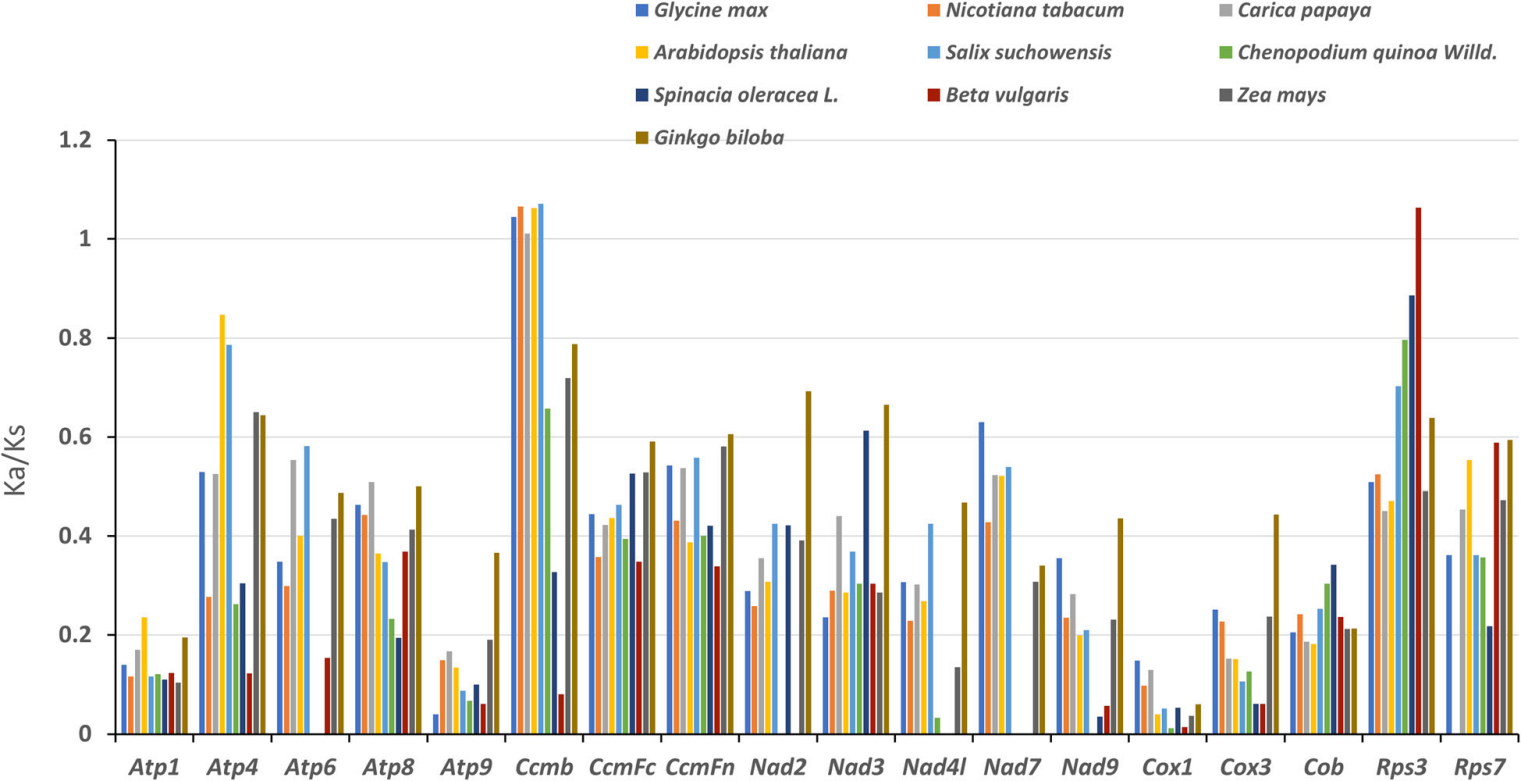
The Ka/Ks values of 18 protein-coding genes of *S. glauca* versus ten species

The calculation of non-synonymous substitutions (Ka) and synonymous substitutions (Ks) is of great significance for the reconstruction of phylogeny and the understanding of evolutionary dynamics of protein-coding sequences in closely related species [36]. In genetics, Ka/Ks value could be used to determine whether selective pressure existed on a specific protein-coding gene during evolution: Ka/Ks > 1, positive selection; Ka/Ks = 1, neutral selection; and Ka/Ks < 1, negative selection [37]. The 18 protein-coding genes from *S. glauca* mt genome were compared with the mt genomes of 10 species, *A. thaliana* (NC_037304), *B. vulgaris* (NC_015099), *C. papaya* (NC_012116)*, G. max* (NC_020455), *S. suchowensis* (NC_029317), Z. mays (NC_008332), *C. quinoa Willd* (NC_041093), *S. oleracea* (NC_035618), *N. icotiana tabacum* (NC_006581), and *G. biloba* (NC_027976) for Ka/Ks calculation. As shown in Fig. 6, the Ka/Ks values of *S. glauca ccmB* compared to *G. max, S. suchowensis, A. thaliana, N. tabacum*, and *C. papaya* were higher than 1, suggesting a positive selection occurred during evolution. However, the Ka/Ks values of most proteins in *S. glauca* were less than 1 compared to the other plant species, indicating the negative selections of those genes during evolution. Taken together, we conclude that the mt genes are highly conserved during the evolutionary process in green plants.

## Discussion

Mitochondria are the powerhouse of the plants that produce the required energy to carry out life processes. Plant mitochondria possess more complex genomes than animals, with extensive size variations, sequence arrangements, repeat content, and a highly conserved coding sequence [38]. Understanding the mt genome structure is required to unravel its function, replication, inheritance, and evolutionary trajectories [38]. In the current study, we studied the characteristics of the mt genome of *S. glauca,* a crucial salt tolerance plant with great value as a food source and phytoremediation agent. According to the reported data, most of the mt genome is circular, and few mt genomes are linear such as the mt genome of *Polytomella parva* [39, 40]. The mt genome of *S. glauca* reported in this study is circular with 474,330 bp in size.

The repeat sequences widely exist in the mt genome, and these repeats include tandem, short, and large repeats [41, 42]. Previous studies have shown that repeats in mitochondria are vital for intermolecular recombination. For this reason, the repeat sequences play a pivotal role in shaping the mt genome [43]. In this study, the SSRs, longer tandem repeats, and non-tandem repeats were intensively investigated (Fig. 2). The mt genome of *S. glauca* harbors abundant repeat sequences that might indicate that the intermolecular recombination frequently happens in the mt genome, which dynamically changes the sequence and conformation during the evolution. We also investigated the genome structure and organization of *S. glauca* in comparison with other land plants. Conclusively, the mt genome characteristics of *S. glauca* were consistent with those of other terrestrial green plants.

RNA-editing is a posttranscriptional process that occurs in the cp and mt genomes of higher plants, contributing to the better folding of proteins [44]. Investigating the RNA-editing sites helps to understand the gene expression of the cp and mt genes in plants. Previous studies reported approximately 441 RNA-editing sites within 36 genes in *Arabidopsis* and 491 RNA-editing sites within 34 genes in rice [39, 45]. In this study, 216 RNA-editing sites within 26 genes were identified. The identification of RNA editing sites provides essential clues for predicting gene functions with novel codons. As the cytoplasmic genome, migration of cp DNA to the mt genome occurred during the plant evolution. We found that 32 fragments were transferred from the cp genome to mt with 8 integrated genes, which are all tRNA genes (Table 5). Transfer of tRNA genes from cp to mt is common in angiosperms [44].

Further, we have analyzed the phylogenetic relationship of *S. glauca* with representative taxa based on the mt genome information. The resulted phylogenetic tree reflected a clear taxonomic relationship among the taxa. We also analyzed GC content of the mt genome in *S. glauca* along with other green plants. The result supports the conclusion that GC content is highly conserved in higher plants. The Ka/Ks analysis and the comparison of genome features with other plant’s mt genomes provide a comprehensive understanding of plant mt evolution. Generally, most of the results in this study were consistent with previous reports. The genes that undergone neutral and negative selections were also identified in *S. glauca*. However, most of the protein-coding genes in *S. glauca* mt had negative selection compared with other selected species, which is consistent with the previous studies, indicating that the protein-coding genes in the mt genome are conserved across the land plants. The *ccmB* gene is the only gene that underwent positive selection during the evolution.

In crop plants, deciphering and understanding the mt genome is essential for plant breeding. Understanding of mt genome will set a foundation for the evolutionary analysis, cytoplasmic male sterility, and molecular biological information for plant breeding. Even though *S. glauca* is not a crop plant, its biological significance and edible values are being examined. As a halophytic model plant with prominent salt-tolerance, whose mt genome has not been reported, the accomplishment of the mt genome provides an opportunity to conduct further genomic studies in *S. glauca*. Therefore, our study provides essential background information for future understanding of this plant [44].

## Conclusion

In this study, we assembled and annotated the mt genome of *S. glauca* and performed extensive analyses based on the DNA sequences and amino acid sequences of the annotated genes. The *S. glauca* mt genome is circular, with a length of 474,330 bp. 61 genes, including 27 protein-coding genes, 29 tRNA genes, and 5 rRNA genes, were annotated in the genome. The repeats sequences and RNA editing in *S. glauca* mt genome were analyzed subsequently. The gene conversation between mt and cp genome was also observed in *S. glauca* by detecting gene migration. Moreover, our result also indicates consistency in molecular and taxonomic classification, besides GC contents in angiosperms, were also found conserved despite their genome sizes that varied tremendously. The Ka/Ks analysis based on code substitution revealed that most of the coding genes had undergone negative selections, indicating the conservation of mt genes during the evolution. This study provides extensive information about the mt genome for *S. glauca*, facilitating deciphering the salt resistance mechanism in plants.

## Methods

### Plant growth conditions, DNA extraction, and sequencing

The *S. glauca* seeds were provided by Chunyin Zhang (Yancheng Lvyuan Salt Soil Agricultural Technology Co. Ltd., Yancheng, Jiangsu, Southeast China, http://www.ychpz.com/index.asp). Seeds were treated with 0.03% Gibberellin for 24 h and germinated at 25 °C in a growth chamber. The seedlings were planted at 25 °C in the greenhouse with 16/8 h of light-dark photoperiod cycle. Leaves from about 40 days old plants were used for DNA isolation using CTAB method [46]. The DNA sample quality was examined with agarose-gel electrophoresis, and the concentration was measured using Nanodrop instrument (2000c UV-Vis). The qualified samples were sent to the Annoroad Gene Technology (http://www.annoroad.com/) for Pacbio sequencing.

### Assembly and annotation of the mitochondrial genome

The mitochondrial sequences of *S. glauca* were selected with blast software using the conserved mitochondrial sequences of *Beta vulgaris*, *Spinacia oleracea*, and *Chenopodium quinoa Willd* as queries. The mt genome was assembled using Canu v1.8 with the selected reads [47]. The assembled contigs were polished (Pilon v 1.18) with Illumina reads to correct read errors. The GE-Seq tool on MPI-MP CHLOROBX website [48] (https://chlorobox.mpimp-golm.mpg.de) was used for the mt genome annotation using the mt genomes of the following species as references: *Arabidopsis thaliana* (NC_037304), *Beta vulgaris* (NC_002511), *Brassica napus* (NC_008285), *Carica papaya* (NC_012116), *Chenopodium quinoa Willd* (NC_041093), *Daucus carota* (NC_017855), *Glycine max* (NC_020455), *Nicotiana tabacum* (NC_006581), *Spinacia oleracea.* (NC_035618), and *Salix suchowensis* (NC_029317) as references. The threshold for protein search identity was 55%, and that of rRNA, tRNA, and DNA search identity was 85%. The annotation results from Ge-Seq were manually adjusted with Mega 7.0 [49]. The output genebank format file was manually confirmed, and the mitochondrial circular map was drawn using Organellar Genome DRAW (OGDRAW) [50].

### Analysis of repeated sequences

Microsatellite identification tool was used to detect simple sequence repeats [51] (https://webblast.ipk-gatersleben.de/misa/index.php). The repeats of 1, 2, 3, 4, 5, and 6 bases with 8, 4, 4, 3, 3, and 3 repeats numbers, respectively, were identified in this analysis. The tandem repeats with > 6p repeat unit were detected using Tandem Repeats Finder v4.09 software [28] (http://tandem.bu.edu/trf/trf.submit.options.html) with default parameters. The direct and inverted repeats were detected using REPuter software [30] (https://bibiserv.cebitec.uni-bielefeld.de/reputer) with the minimal repeat size set to 20 bp.

### Chloroplast to mitochondrion DNA transformation and RNA editing analyses

DNA migration is common in plants and varies from species to species [52]. This phenomenon occurs during autophagy, gametogenesis, and fertilization [53]. The cp genome of *S. glauca* (NC_045302.1) was downloaded from NCBI Organelle Genome Resources Database. Blastn software on NCBI was used to identify the protein-coding and tRNA genes transferred from chloroplasts to mitochondria. Screening criteria were set as the matching rate ≥ 70%, E-value ≤ 1e ^− 10^, and length ≥ 40. The editing sites in the mitochondrial RNA of *S. glauca* were revealed using the mt gene encoding proteins of plants as references. The analysis was conducted on the Plant Predictive RNA Editor (PREP) suite [34] (http://prep.unl.edu/) with a cut off value of 0.2.

### Phylogenetic tree construction and Ka/Ks analysis

The conserved protein-coding genes from mt genomes of *S. glauca* and other 28 taxa were used for phylogenetic tree construction. The mt genomes were downloaded from NCBI, and the conserved protein-coding genes (*atp1, atp4, atp6, atp8, atp9, ccmB, ccmC, ccmFc, ccmFn, cob, cox1, cox2, cox3, matR, nad1, nad2, nad3, nad4L, nad5, nad6, nad7, and nad9*) were extracted using TBtool software [54], and then aligned using Muscle software [55]. Subsequently, a Neighbor-joining (NJ) tree was constructed by Mega 7.0 software using the Poisson model with a bootstrap of 1000 [49]. *C. taitungensis* and *G. biloba* were designated as the outgroup in this analysis. The synonymous (Ks) and non-synonymous (Ka) substitution rates of the protein-coding genes in *S. glauca* mt genome were analyzed using ten representative species (Table S3) as references. In this analysis, Mega 7.0 [49] was used for sequence alignment, and DNAsP v.6.12 [56] was used to calculate Ka/Ks.

## Supplementary Information

**Additional file 1: Figure S1**. The secondary structure of tRNA. A and B are two different structures of *trnM-CAU*. **Figure S2.** The distribution of SSRs in *S. glauca* mt genome. The colors represent different types of SSRs. The area on the pie chart indicates the percentages of different SSR types. **Table S1.** The mt homologous genes in *S. glauca*, *A. thaliana*, *H. sapiens*, and *S. cerevisiae*. **Table S2.** The stop codes of protein-coding genes in *S. glauca* mt genome. **Table S3.** The abbreviations and NCBI accession numbers of mt genomes used in this study. **Table S4.** Protein-coding genes annotated in *S. gluaca* mt genome in comparison to related species.

**Additional file 2: **The sequence and annotation of *S. glauca* mt genome.

**Additional file 3: Additional data sheet 1.** The distribution of repeats in the *S. glauca* mt genome**.**

## Data Availability

The sequence and annotation of *S. glauca* mt genome was provided as Additional file 2. The accession number in Gene Banks is MW561632.

## Abbreviations

*S. glauca*: *Suaeda glauca*
mt: mitochondria
cp: chloroplast
