# Supplementary material for: Assembly and comparative analysis of the complete mitochondrial genome of Suaeda glauca
Source: BMC Genomics. 2021 Mar 9;22:167. doi: 10.1186/s12864-021-07490-9 (PMC7941912; doi:10.1186/s12864-021-07490-9)
Supplement: Supplementary file 1 — Additional file 1: Figure S1. The secondary structure of tRNA. A and B are two different structures of trnM-CAU. Figure S2. The distribution of SSRs in S. glauca mt genome. The colors represent different types of SSRs. The area on the pie chart indicates the percentages of different SSR types. Table S1. The mt homologous genes in S. glauca, A. thaliana, H. sapiens, and S. cerevisiae. Table S2. The stop codes of protein-coding genes in S. glauca mt genome. Table S3. The abbreviations and NCBI accession numbers of mt genomes used in this study. Table S4. Protein-coding genes annotated in S. gluaca mt genome in comparison to related species. [file 12864_2021_7490_MOESM1_ESM.pdf]

Supplementary Figures and Tables

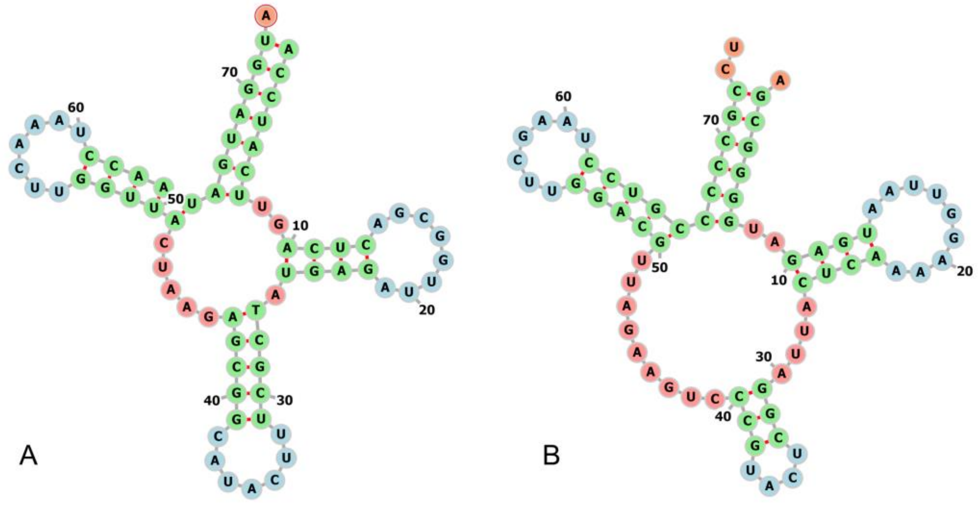

Figure S1. The secondary structure of tRNA. A and B are two different structures of *trnM-CAU*.

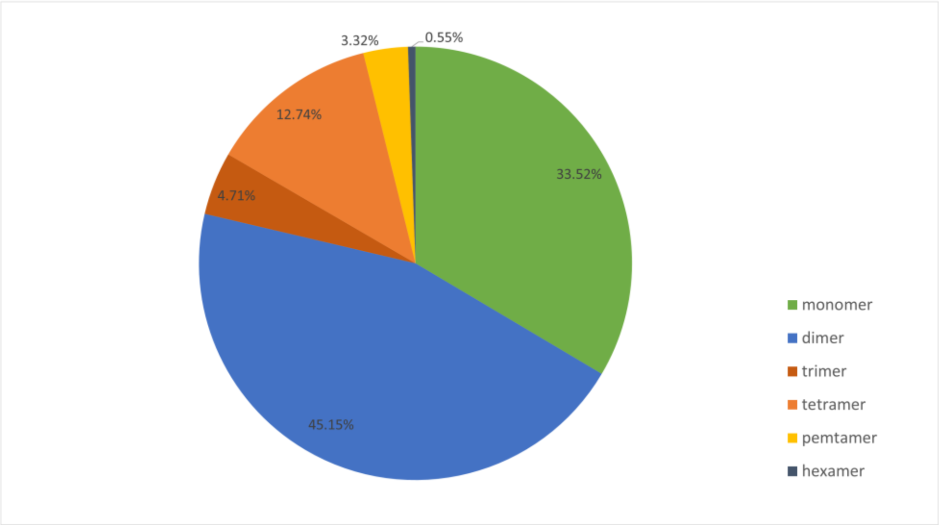

Figure S2. The distribution of SSRs in *S. glauca* mt genome. The colors represent different types of SSRs. The area on the pie chart indicates the percentages of different SSR types.

Table S1. The mt homologous genes in *S. glauca*, *A. thaliana*, *H. sapiens*, and *S. cerevisiae*.

	S. glauca	*A. thaliana*	*H. sapiens*	*S. cerevisiae*
NADH dehydrogenase	*nad1*	*nad1*	*ND1*	-
	nad2	*nad2*	*ND5*	-
	nad3	*nad3*	*ND3*	-
	nad4L	*nad4L*	-	-
	nad5	*nad5*	*ND5*	-
	nad7	*nad7*	-	-
	nad9	*nad9*	-	-
ATP synthase	*atp1*	*atp1*	-	-
	atp4	*atp4*	-	-
	atp6	*atp6*	-	-
	atp8	*atp8*	-	-
	atp9	*atp9*	-	-
Cytochrome c biogenesis	*ccmB*	*ccmB*	-	-
	ccmC	*ccmC*	-	-
	ccmFC	*ccmFC*	-	-
	ccmFN	*ccmFN2*	-	-
Cytochrome c oxidase	*cox1*	*cox1*	-	-
	cox2[a]	*cox2*	*Cox2*	*Cox2*
	cox3	*cox3*	*Cox3*	*Cox3*
Maturases	*matR*	*matR*	-	-
Ubiquinol cytochrome c reductase	*cob*	*cob*	*CYTB*	*cob*
Ribosomal proteins (LSU)	*rpl5*	*rpl5*	-	-
Ribosomal proteins (SSU)	*rps3*	*rps3*	-	-
	rps7	*rps7*	-	-
	rps12	*rps12*	-	-
Transport membrane protein	*sdh4*	*sdh4*	-	-
Ribosomal RNAs	*rrn5*	*rrn5*	-	-
	rrnS	*rrn18*	-	-
	rrnL(3)	*rrn26*	-	-
Transfer RNAs	*trnA-UGC*	-	-	-
	trnC-GCA	*trnC-GCA*	-	-
	trnE-UUC	*trnE(UUC)*	-	-
	trnF-GAA	-	-	-
	trnG-GCC	*trnG(GCC)*	-	-
	trnH-GUG	*trnH(GUG)*	-	-
	trnI-GAU	*trnN(GUU)*	-	-
	trnK-UUU	*trnK(UUU)*	-	-
	trnL-CAA	-	-	-
	trnM-CAU	*trnM(CAU)*	-	-
	trnN-GUU	*trnN(GUU)*	-	-
	trnP-UGG	*trnP(UGG)*	-	-
	trnQ-UUG	*trnQ(UUG)*	-	-
	trnR-ACG	-	-	-
	trnS-GCU	*trnS(GCU)*	-	-
	trnS-UGA	*trnS(UGA)*	-	-
	trnV-GAC	*trnN(GUU)*	-	-
	trnW-CCA	*trnW(CCA)*	-	-
	trnY-GUA	*trnY(GUA)*	-	-

Note: "-" indicate the absence of the specific gene.

Table S2. The stop codes of protein-coding genes in *S. glauca* mt genome.

Stop codon	Number	Percentage
TAG	5	18.52%
TGA	10	37.04%
TAA	12	44.44%
Total	27	100%

Table S3. The abbreviations and NCBI accession numbers of mt genomes used in this study.

Species	Abbreviations	Accession Numbers
Arabidopsis thaliana	*A. thanliana*	NC_037304
Bartramia pomiformis	*B. pomiformis*	NC_024519
Beta vulgaris	*B. vulgaris*	NC_002511
Brassica napus	*B. napus*	NC_008285
Buxbaumia aphylla	*B. aphylla*	NC_024518
Capsicum annuum	*C. annuum*	NC_024624
Chlamydomonas reinhardtii	*C. reinhardtii*	NC_001638
Chlorella heliozoae	*C. heliozoae*	KY629615
Chara vulgaris	*C. vulgaris*	NC_005255
Carica papaya	*C. papaya*	NC_012116
Citrullus lanatus	*C. lanatus*	NC_014043
Cucumis sativus	*C. sativus*	NC_016005
Cucurbita pepo	*C. pepo*	NC_014050
Cycas taitungensis	*C. taitungensis*	NC_010303
Daucus carota	*D. carota*	NC_017855
Ginkgo biloba	*G. biloba*	NC_027976
Glycine max	*G. max*	NC_020455
Homo sapiens	*H. sapiens*	NC_012920
Lotus japonicas	*L. japonicas*	NC_016743
Malus domestica	*M. domestica*	NC_018554
Millettia pinnata	*M. pinnata*	NC_016742
Marchantia palealea	*M. palealea*	NC_001660
Medicago truncatula	*M. truncatula*	NC_029641
Nicotiana tabacum	*N. tabacum*	NC_006581
Nitella hyalina	*N. hyalina*	NC_017598
Oryza sativa	*O. sativa*	NC_007886
Populus tremula	*P. tremula*	NC_028096
Raphanus sativus	*R. sativus*	NC_018551
Rhazya stricta	*R. stricta*	NC_024293
Saccharomyces cerevisiae	*S. cerevisiae*	NC_001224
Salix suchowensis	*S. suchowensis*	NC_029317
Sorghum bicolor	*S. bicolor*	NC_008360
Sphagnum palustre	*S. palustre*	NC_024521
Triticum aestivum	*T. aestivum*	NC_007579
Vigna angularis	*V. angularis*	NC_021092
Vitis vinifera	*V. vinifera*	NC_012119
Zea mays	*Z.mays*	NC_008332
Chenopodium quinoa	C. *quinoa*	NC_041093
Spinacia oleracea	*S. oleracea*	NC_035618

Table S4. Protein-coding genes annotated in *S. gluaca* mt genome in comparison to related species.

Gene	*B. vulgaris*	*S. glauca*	*C. quinoa*	*S. oleracea*
atp1	+	+	+	+
atp4	-	+	+	+
atp6	+	+	+	+
atp8	+	+	+	+
atp9	+	+	+	+
ccmB	+	+	+	+
ccmC	-	+	+	+
ccmFc	+	+	+	+
ccmFn	+	+	+	+
cob	+	+	+	+
cox1	+	+	+	+
cox2	+	+	+	+
cox3	+	+	+	+
matR	+	+	+	+
nad1	++	+	+	-
nad2	+	+	+	+
nad3	+	+	+	+
nad4	+	-	+	+
nad4L	+	+	+	+
nad5	+	+	+	+
nad6	+	-	+	+
nad7	+	++	+	+
nad9	+	+	+	+
rpl5	+	+	+	+
rps3	+	+	+	+
rps4	+	-	+	+
rps12	+	+	+	+
rps13	+	-	+	+
rps7	++	+	+	+
sdh4	+	+	-	-
tatC	+	-	+	+

Note: ‘+’ indicates the presence of the gene in the specific species, and each ‘+’ represents one copy number. ‘−’ indicates the absence of the specific gene.
